# Rapid detection of *ERG11 *gene mutations in clinical *Candida albicans *isolates with reduced susceptibility to fluconazole by rolling circle amplification and DNA sequencing

**DOI:** 10.1186/1471-2180-9-167

**Published:** 2009-08-14

**Authors:** Huiping Wang, Fanrong Kong, Tania C Sorrell, Bin Wang, Paul McNicholas, Namfon Pantarat, David Ellis, Meng Xiao, Fred Widmer, Sharon CA Chen

**Affiliations:** 1 Department of Dermatology, Tianjin Medical University General Hospital, Tianjin Medical University, Tianjin, PR China; 2 Centre for Infectious Diseases and Microbiology, The University of Sydney, Westmead Hospital, Sydney, Australia; 3 Retroviral Genetics Laboratory, Centre for Virus Research, Westmead Millennium Institute and the University of Sydney, Sydney, Australia; 4 Schering-Plough Research Institute, Kenilworth, New Jersey, USA; 5 Mycology Unit, Women's and Children's Hospital, Adelaide, Australia; 6 Life Science College, Peking University, Beijing, PR China

## Abstract

**Background:**

Amino acid substitutions in the target enzyme Erg11p of azole antifungals contribute to clinically-relevant azole resistance in *Candida albicans*. A simple molecular method for rapid detection of *ERG11 *gene mutations would be an advantage as a screening tool to identify potentially-resistant strains and to track their movement. To complement DNA sequencing, we developed a padlock probe and rolling circle amplification (RCA)-based method to detect a series of mutations in the *C. albicans ERG11 *gene using "reference" azole-resistant isolates with known mutations. The method was then used to estimate the frequency of *ERG11 *mutations and their type in 25 Australian clinical *C. albicans *isolates with reduced susceptibility to fluconazole and in 23 fluconazole-susceptible isolates. RCA results were compared DNA sequencing.

**Results:**

The RCA assay correctly identified all *ERG11 *mutations in eight "reference" *C. albicans *isolates. When applied to 48 test strains, the RCA method showed 100% agreement with DNA sequencing where an *ERG11 *mutation-specific probe was used. Of 20 different missense mutations detected by sequencing in 24 of 25 (96%) isolates with reduced fluconazole susceptibility, 16 were detected by RCA. Five missense mutations were detected by both methods in 18 of 23 (78%) fluconazole-susceptible strains. DNA sequencing revealed that mutations in non-susceptible isolates were all due to homozygous nucleotide changes. With the exception of the mutations leading to amino acid substitution E266D, those in fluconazole-susceptible strains were heterozygous. Amino acid substitutions common to both sets of isolates were D116E, E266D, K128T, V437I and V488I. Substitutions unique to isolates with reduced fluconazole susceptibility were G464 S (n = 4 isolates), G448E (n = 3), G307S (n = 3), K143R (n = 3) and Y123H, S405F and R467K (each n = 1). DNA sequencing revealed a novel substitution, G450V, in one isolate.

**Conclusion:**

The sensitive RCA assay described here is a simple, robust and rapid (2 h) method for the detection of *ERG11 *polymorphisms. It showed excellent concordance with *ERG11 *sequencing and is a potentially valuable tool to track the emergence and spread of azole-resistant *C. albicans *and to study the epidemiology of *ERG11 *mutations. The RCA method is applicable to the study of azole resistance in other fungi.

## Background

*Candida albicans *causes systemic infections, typically in immunocompromised patients, as well as mucosal infections such as oropharyngeal candidiasis (OPC) in HIV-infected patients and chronic vaginal infections [1,2]. Azole antifungal drugs are the mainstay of management of such infections. However, with increased use of these agents, particularly fluconazole, treatment failures associated with the emergence of azole-resistant strains of *C. albicans *have occurred [3-6] This has been most evident in HIV/AIDS patients receiving long-term therapy for OPC [3,7]

The azoles bind to and inhibit the activity of lanosterol 14α-demethylase (Erg11p), a key enzyme in the fungal ergosterol biosynthesis pathway [8]. Several mechanisms of resistance to azoles have been described in *C. albicans*. These include increased expression of the drug efflux pump genes such as *MDR1, CDR1 *and *CDR2 *[3,9-11], amino acid substitutions in the target enzyme Erg11p due to missense mutations in the *ERG11 *gene [3,5,10,12-15] and possibly, overexpression of *ERG11 *[3,16] Importantly in any one isolate, resistance may be due to a combination of mechanisms [3-5,15].

To date, more than 60 amino acid substitutions have been described in Erg11p with at least 30 of these identified in azole-resistant isolates [5,12,14-17] The impact of individual substitutions, however, varies, and may differ between azoles. For example, substitutions such as Y132H, G450E, G464S, R467K and S405F appear to primarily impact on fluconazole and voriconazole, but not posaconazole, susceptibility [12,13,16,17] The effect of substitutions can also be additive – strains with G129A and G464S substitutions display higher MICs azoles compared with those with the G129A substitution alone [18]. The contributions of yet other *ERG11 *mutations to resistance are uncertain [15,19]. As most strains of *C. albicans *are diploid, nucleotide mutations may occur as homozygous (in both alleles) or as heterozygous (in one allele) substitutions; the association of either type of mutation with the resistant (or susceptible) phenotype is not well defined [3,20].

**Figure 1:**
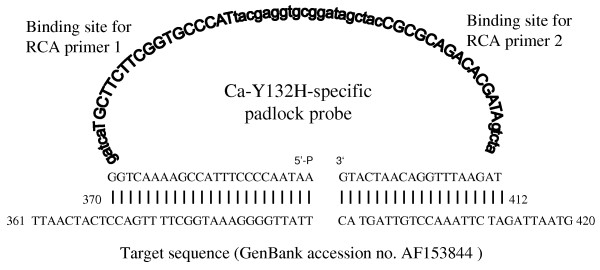
**Typical design of a circularisable padlock probe**. Design and components of a typical padlock probe as exemplified by the Ca-Y132H probe specific for the Y132H amino acid substitution. The probe comprises (i) a 5'-phosphorylated end; (ii) a "backbone" containing binding sites for the RCA primers (RCA primer 1 and 2, respectively) designated by bold upper case letters) as well as the non-specific linker regions (designated by bold lower case letters) and (iii) a 3'-end. The 5'- and 3'-ends of the probe are complementary to the 5' and 3' termini of the target sequence in reverse, in this example, to the *C*. *albicans *sequence (GenBank accession no. AF153844). Abbreviations: 5'-P, 5'-phosphorylated binding arm; 3'-, 3' binding arm.

Nonetheless, since even single nucleotide polymorphisms (SNPs) in the *ERG11 *gene can have an impact on susceptibility and contribute to resistance [5,13,15,18,19,21], the documentation of these changes is important. To date, the frequency and clinical relevance of specific mutations in unselected azole-resistant isolates is poorly-defined [17] although in one survey, *ERG11 *mutations contributed to resistance in 65% of fluconazole-resistant *C. albicans *from HIV patients with OPC [5]. In clinical practice, detection of *ERG11 *mutations as potential markers or co-markers of resistance would assist both the identification and tracking of azole-resistant strains. Traditionally, DNA sequence analysis has been the standard for identifying *ERG11 *nucleotide changes [5,14,17,19] However, circularisable or padlock probes have recently been shown to reliably detect SNPs with high specificity, offering a rapid simple alternative to sequencing [22,23]. Padlock probes comprise three distinct regions: a central linker is flanked by sequences complementary to the 5' and 3' termini of the target sequence. Upon hybridisation to the target, the probe ends are brought together and are joined by DNA ligase to form a closed circular molecule in a highly target-dependent manner (Figure 1). The intensity of the probe signal is then increased exponentially by rolling circle amplification (RCA) generating up to a 10^9^-fold signal amplification within 90 min [22-24] RCA-based assays have been successfully used to identify fungal pathogens [25,26] but have not yet been applied to the detection of gene mutations associated with antifungal drug resistance.

The present report describes the development and validation of a sensitive RCA-based SNP detection assay using real time PCR to detect point mutations in the *C. albicans ERG11 *gene in eight azole-resistant "reference" isolates with known mutations [15]; *ERG11 *was chosen as the target gene to detect SNPs associated with azole resistance in a proof of principle study. In the study, RCA and DNA sequencing were applied to estimate the frequency of a series of *ERG11 *mutations and their nature in 25 unselected Australian *C. albicans *clinical isolates with reduced susceptibility to fluconazole. The results were compared with those obtained for 23 fluconazole-susceptible strains.

## Results

### *C. albicans *isolates and azole susceptibilities

Thirty-three isolates had reduced susceptibility to fluconazole. Twenty-eight were recovered from the oropharynx, two from the vagina and one each from bile, sputum and blood (Tables 1 and 2). The *ERG11 *gene from eight of these isolates, referred to as "reference" isolates, was previously sequenced (see Methods and Table 1) [15]; the remaining 25 clinical isolates were interrogated for *ERG11 *mutations (Table 2). An additional 23 fluconazole-susceptible isolates (see Methods for categories of susceptibility/resistance) cultured from a range of body sites (Table 2) were studied. Thus 48 "test" isolates were analysed by RCA and DNA sequencing.

**Table 1:** RCA^a^**analysis of azole–resistant *C. albicans *isolates with known *ERG11 *mutations**^b^.

			MIC (μg/ml)			
					
**Patient no**.	Isolate **no**.	Body site of isolation	FLU^a^	VOR^a^	Previously-characterized amino acid substitution(s)	Erg11p substitution(s) by RCA
1	C438	Oropharynx	128	2	Y257H, G464S	Y257H, G464S
	C440	Oropharynx	>256	>16	A61V, Y257H, G307S, G464S	A61V, Y257H, G307S, G464S
2	C470	Oropharynx	32	0.25	S405F	S405F
3	C480	Oropharynx	128	8	G464S, K128T, R467I	G464S, K128T, R467I
4	C507	Oropharynx	64	8	G464S, H283R, Y132H	G464S, H283R, Y132H
5	C527	Oropharynx	256	4	G450E, Y132H	G450E, Y132H
6	C577	Oropharynx	128	0.5	G464S	G464S
7	C594	Oropharynx	128	16	S405F, Y132H	S405F, Y132H

^a ^Abbreviations: RCA, rolling circle amplification; FLU, fluconazole; VOR, voriconazole.

^b ^Chau *et al*. [15].

**Table 2:** MIC results and Erg11p substitutions for 25 *C. albicans *isolates with reduced susceptibility to fluconazole and 23 fluconazole-susceptible isolates by RCA^a ^and *ERG11 *sequencing.

		MIC (μg/ml)		Erg 11p amino acid substitutions															
				D	D	E	F	F	G	G	G	G	K	K	R	S	V	V	Y
				1	2	2	1	4	3	4	4	4	1	1	4	4	4	4	1
**Patient/isolate no**.	Site	FLU^a^	VOR^a^	1	7	6	4	4	0	4	6	6	2	4	6	0	3	8	3
				6	8	6	5	9	7	8	4	5	8	3	7	5	7	8	2
				E	E	D	L	S	S	E	S	S	T	R	K	F	I	I	H
***Isolates with reduced fluconazole susceptibility***																			
1^b^	Oropharynx	16	0.25			+	+											+	
2^b^	Vagina	>256	0.03																
3-A^b, c^	Oropharynx	16	0.25			+	+											+	
- B^b, c^	Oropharynx	16	0.5			+	+											+	
4^d^	Oropharynx	256	0.25			+								+				+	
5^d^	Oropharynx	256	0.125						+										
6-A^c, d^	Oropharynx	256	>16			+													
-B^c, d^	Oropharynx	256	>16	+															
7^d^	Oropharynx	256	>16			+					+			+				+	
8-A^c, d^	Oropharynx	256	0.5			+		+										+	
-B^c, d^	Oropharynx	256	1			+		+										+	
9^d^	Oropharynx	256	>16			+											+		
10^d^	Oropharynx	256	2			+										+		+	+
11^d^	Oropharynx	256	0.008			+											+		
12-A^c, d^	Oropharynx	256	2								+								
-B^c, d^	Sputum	32	0.5	+							+	+							
13^d^	Vagina	32	0.016										+						
14^d^	Blood	256	>16	+									+						
15^d^	Bile	256	16														+		
16-A^c, d^	Oropharynx	16	0.125	+						+			+						
-B^c^	Oropharynx	256	2	+					+	+			+						
-C^c, d^	Oropharynx	256	16	+					+	+	+		+						
17^d^	Oropharynx	64	0.5		+														
18^d^	Oropharynx	128	2	+									+		+				
19^d^	Oropharynx	256	0.5	+									+	+					
***Fluconazole-susceptible isolates*^b^**																			
ATCC 10231	UN^a^	0.125	0.008																
ATCC 90028	Blood	025	0.03																
20	Blood	0.25	<0.008			+											+		
21	Oropharynx	0.12	0.008	+		+													
22	Blood	0.5	0.016																
23	Blood	0.5	0.016																
24	Blood	0.25	0.008																
25	Blood	0.25	0.008			+												+	
26	Blood	0.5	0.008	+		+													
27	Blood	0.5	0.008	+		+													
28	Blood	0.25	<0.008			+											+		
29	Skin	1	0.016	+									+						
30	Peritoneal fluid	1	2	+		+													
31	Blood	0.12	0.125	+		+												+	
32	Blood	0.125	0.008	+									+						
33	Blood	0.5	0.008	+		+												+	
34	Tissue	0.25	0.008	+									+						
35	Blood	2	0.008			+												+	
36	Blood	0.25	0.016			+												+	
37	Liver	0.5	<0.008			+												+	
38	Blood	0.25	<0.008	+		+												+	
39	Blood	0.125	<0.008			+													
40	Bone	0.25	<0.008	+		+													

The "+" sign denotes the presence of the mutation.

^a^Abbreviations: RCA, rolling circle amplification; FLU, fluconazole; VOR, voriconazole; UN, unknown.

^b ^Isolates from the Centre for Infectious Diseases and Microbiology, Westmead Hospital, Sydney.

^c ^The "A" and "B" notation of patient numbers refers to isolates cultured sequentially from the same patient at different times.

^d^Isolates obtained from the Mycology Unit, Women's and Children's Hospital, Adelaide.

One of the eight "reference" isolates was susceptible-dose dependent (S-DD; MIC 16–32 μg/ml) to fluconazole and seven were fluconazole-resistant (MIC ≥ 64 μg/ml; Table 1); five of these seven were also resistant to voriconazole (MIC ≥ 4 μg/ml) [15,27]. Six of the 25 Australian isolates (from patients 1, 3, 12, 13 and 16; Table 2) had fluconazole MICs in the S-DD range and were susceptible to voriconazole; the remaining 19 were resistant to fluconazole and seven (from patients 6, 7, 9, 14, 15 and 16) of these were cross-resistant to voriconazole (Table 2). All 23 fluconazole-susceptible isolates were also susceptible to voriconazole (Table 2).

### Detection of ERG11 mutations by RCA: Sensitivity and specificity

**Figure 2:**
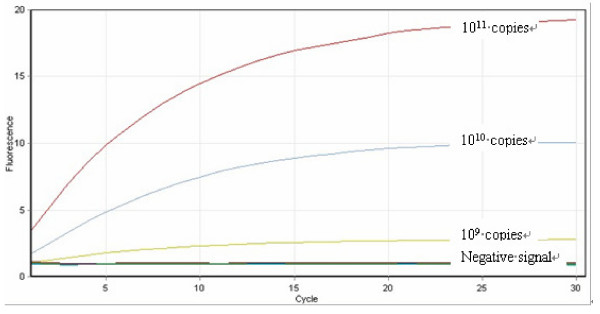
**Sensitivity of the RCA assay**. RCA was performed on 10-fold serial dilutions of the target template ranging from 10^11 ^to 10^0 ^copies of target template (PCR product). The figure illustrates the RCA reaction using the Ca-Y132H-specific probe to detect 10^11^, 10^10 ^and 10^9 ^copies of the template containing the Y132H mutation (obtained from amplifying DNA from isolate C594). RCA signals are shown as exponential increases in florescence signal above baseline (indicated by the "negative signal" label and defined as the signal obtained when amplifying target template that did *not *have the mutation of interest). The intensity of the signal weakened with decreasing copy numbers starting at 10^11^copies and the sensitivity of the assay corresponded to a concentration of 10^9^copies of target template.

Point mutation-specific RCA probes were designed based on the target polymorphism site of interest in the *ERG11 *gene (see Additional file 1). To assess the sensitivity of the RCA-based assay, RCA was initially performed on 10-fold serial dilutions of the target template (PCR product; see Methods) ranging from 10^11 ^to 10^0 ^copies of template. For all isolates studied, a clear RCA fluorescence signal was observed with a sensitivity of detection of 10^9 ^copies; below this copy number, the signal was not easily distinguishable from the background signal (as defined when amplifying target template that did *not *have the mutation of interest) (Figure 2). Only signals that were clearly measurable above background were considered to be indicative of the presence of the mutation.

**Figure 3:**
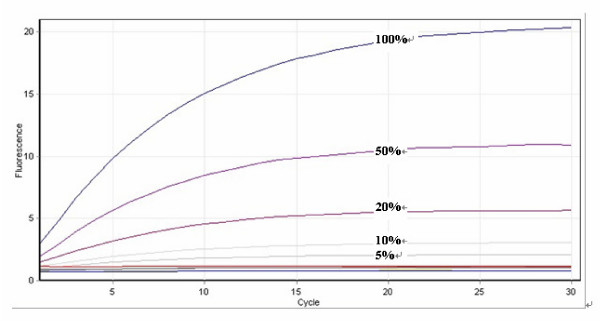
**Sensitivity of the RCA assay in the presence of DNA mixtures**. The accumulation of double-stranded DNA was detected by staining with Sybr green I. RCA signals indicative of the presence of a specific mutation are shown as exponential increases in fluorescence signal above baseline. The figure illustrates the padlock probe-RCA reaction using the Ca-Y257H-specific probe to detect varying concentrations (100%, 50%, 20%, 10% and 5%) of target template (10^11^copies). The target template was DNA from isolate C594 containing the Y257H mutation; this was diluted with DNA from strain ATCC 10231 (without the Y257H mutation). The intensity of RCA fluorescence signal weakened with decreased template concentration. The sensitivity of the assay corresponded to a concentration of 5% template DNA in the mixture.

The capability of the RCA assay to detect heterozygous, as well as homozygous *ERG11 *nucleotide changes was assessed indirectly by testing its ability to detect a specific mutation in the presence of wild-type template (ie. template without the mutation of interest) using the eight "reference" isolates. For each of the known *ERG11 *mutations (Table 1), target template (10^11 ^copies) containing the mutation at 100%, 50%, 20%, 10%, 5%, 2% and 0% concentration in a backdrop of wild-type template were prepared by mixing both templates at the above-mentioned ratios. In all cases, a clear RCA signal above background was observed down to a dilution containing 5% target template (Figure 3); results were reproducible with minimal or no variation in repeat (n = 3) experiments. The results demonstrate that the RCA assay was able to detect *ERG11 *mutations with high sensitivity in the presence of mixtures of DNA and that the sensitivity was well above that required to detect heterozygous nucleotide changes (expected ratio of target template (with mutation) to template without mutation of 1:1)).

**Figure 4:**
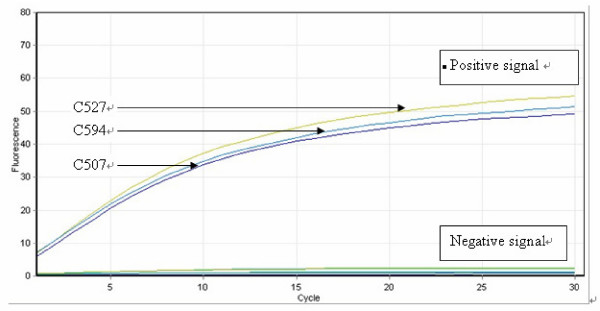
**Specificity of the RCA assay**. RCA results monitored by the RotorGene 6000 real-time PCR machine (Corbett research). The accumulation of double-stranded DNA was detected by staining with Sybr Green I. RCA signals indicating the presence of the mutation of interest ((labeled as "positive signal") are shown as exponential increases in fluorescence. The experiment was conducted using the Ca-Y132H-specific RCA probe and tested on eight *C. albicans *isolates with known *ERG11 *mutation sites (Table 1). Ligation-mediated RCA with matched templates (DNA from isolates C527, C594, C507) containing the targeted SNPs produced "positive signals". Other templates showed an absence of signal (labeled as "negative signal").

The RCA assay was also highly specific. Amplification of probe signals was seen only with matched template-probe mixtures. No signal was seen when template from isolates that did not contain the *ERG11 *polymorphism targeted by a specific padlock probe were used. Figure 4 illustrates a typical padlock probe-RCA reaction using a probe to detect the Erg11p Y132H mutation. For isolates C507, C527 and C594 (Table 1), exponential increases in fluorescence signals were readily interpretable, indicating the presence of the Y132H mutation. Other "reference" isolates produced a signal at "background" level, indicative of absence of the mutation. All 10 known *ERG11 *mutations in the "reference" isolates were correctly identified. The duration of the RCA procedure was 2 h; however, a readily discernible signal was usually evident 15 min after commencement of the RCA reaction.

### Investigation of ERG11 mutations in test isolates by RCA and ERG11 sequencing

The *ERG11 *gene for each of the 48 test isolates (25 non-fluconazole susceptible and 23 fluconazole-susceptible) was amplified by PCR and a 1370 bp fragment (nt 131–1500) was probed using RCA or subject to DNA sequencing (Table 2).

### Isolates with reduced fluconazole susceptibility

By sequencing, all but one isolate (from patient 2; Table 2) contained at least one missense mutation when compared with the *C. albicans *ATCC 28526 sequence (GenBank accession no. AF153844) (results not shown). Results obtained by the RCA assay were concordant with DNA sequencing for all isolates. However, DNA sequencing identified four additional amino acid substitutions (D153E, F126L, K108E and a novel substitution G450V) in six separate isolates (from patients 5, 6, 10, 11 and 12 as shown in Table 3) – these substitutions were not detected by RCA as the corresponding padlock probes targeting these mutation sites were not used in the study. Thus *ERG11 *point mutations resulting in 16 different amino acid substitutions were detected among the 25 test isolates by RCA (Table 2) whereas 20 substitutions were identified by DNA sequencing. Sequencing identified that all amino acid substitutions were due to homozygous nucleotide polymorphisms.

**Table 3:** Additional amino acid substitutions identified by *ERG11 *sequencing in five *C. albicans *isolates with reduced susceptibility to fluconazole.

Patient/isolate no.	Substitutions detected by RCA	Substitutions detected by DNA sequencing
5	G307S	G307S, G450V
6-A^a^	E266D	E266D, D153E
6-B^a^	D116E	D116E, D153E
10	E266D, V488I, S405F, Y132H	E266D, V488I, S405F, Y132H, K108E
11	E266D, V437I	E266D, V437I, F126L

12-A^a^	G464S	G464S, K108E

^a ^The "A" and "B" notation of patient numbers refers to isolates which were cultured sequentially from the same patient at different times.

The substitution G464S was present in four isolates, G448E and G307S were present in three isolates each and the substitutions Y132H, S405F and R467K (each n = 1) were rare (Table 2). Of note, five of the 10 *ERG11 *mutations (leading to amino acid substitutions A61V, G450E, H238R, R467I and Y257H) present in "reference" isolates from the United States (Table 1) were not detected in Australian isolates. Overall, the most frequently-identified substitutions were E266D (n = 11 isolates) followed by V488I (n = 8), D116E (n = 8) and K128T (n = 7). Nineteen of the 20 mutations (95%) were clustered in three regions of Erg11p: positions 105–165, 266–287 and 405–488 (Table 2).

Sequential isolates were available from five patients (patients 3 6, 8, 12 and 16). Isolates from patients 3 and 8 had similar *ERG11 *mutation and MIC profiles; however, isolates from patient 16 demonstrated a step-wise increase in voriconazole MICs in parallel with additional amino acid substitutions; the isolate with the highest MIC contained five substitutions while the isolate with the lowest MIC contained three (Table 2). Conversely, for patient 12, one additional mutation was present from the analysis of the second isolate (isolate 12B; see also Table 3) but the fluconazole and voriconazole MICs of this isolate were lower than that for isolate 12A. Both isolates from patient 6 had similar azole MICs but had one different *ERG11 *mutation (Tables 2 and Table 3).

### Fluconazole-susceptible isolates

No *ERG11 *mutations were detected by either RCA or *ERG11 *sequencing in five of the 23 (22%) fluconazole-susceptible isolates. In the other 18, five amino acid substitutions namely E266D (n = 15 isolates), D116E (n = 11), V488I (n = 7), K128T (n = 3) and V437I (n = 2) were identified (Table 2). By sequencing, homozygous nucleotide changes that led to the E266 substitution were present in all isolates; for the remaining amino acid substitutions, nucleotide mutations were heterozygous. In all cases, RCA results were concordant with those obtained by DNA sequencing confirming that the RCA-based assay is capable of detecting both homozygous and heterozygous SNP substitutions in *ERG11*.

### Mutations unique to isolates with reduced fluconazole susceptibility

Fifteen of the 20 Erg11p amino acid substitutions present in *C. albicans *isolates displaying S-DD susceptibility or resistance to fluconazole were not identified in fluconazole-susceptible strains (Table 2). These included the substitutions G307S, G464S, G448E R467K, S405F and Y132H which have been reported to result in reduced susceptibility to azoles [5,10,15]

## Discussion

Azole antifungals are widely used for therapy and prophylaxis of *Candida *infections. A better understanding of the mechanisms of resistance to these agents as well as early detection of resistance are essential for patient management. Azole resistance is often due to a combination of factors including increased expression of efflux pumps and missense mutations in *ERG11 *[3-5,15]. The latter have been linked to clinically-relevant increases in the MICs, not only to fluconazole, but also to the newer azoles voriconazole and posaconazole [4,5,10,15] This proof of principle study highlights the great potential of a simple rapid (2 h) and highly-specific RCA-based SNP detection assay that can be readily be performed in the clinical laboratory for the detection and/or surveillance for *ERG11 *mutations. Using this method, we identified Erg11p amino acid substitutions in 24 of 25 previously-uncharacterised Australian isolates with reduced susceptibility to fluconazole.

The sensitivity and reproducibility of the RCA assay was established by determining its ability to detect known *ERG11 *mutations in "reference" isolates (Table 1) in comparison with DNA sequencing. The padlock probes designed for this study also accurately identified and distinguished between SNPs within the *ERG11 *genes in the test isolates. These included SNPs that were located close together such as those at nucleotides 1343, 1346 and 1349 corresponding to the amino acid substitutions G448E, F449S and G450E, respectively (Additional file 1). Importantly, identification of *ERG11 *mutations by the RCA assay was concordant with sequencing in all cases where an *ERG11 *mutation-specific probe was used. An additional finding was that even though probes (or pairs of probes) were not designed to detect heterozygous nucleotide substitutions *per se*, the RCA assay detected such changes in isolates containing an *ERG11 *mutation in only one allele, as demonstrated by their identification in fluconazole-susceptible isolates.

A large number (n = 20) of amino acid substitutions were identified in test isolates with reduced susceptibility/resistance to fluconazole. In agreement with a prior report, all but one isolate had at least one, and often multiple missense mutations in *ERG11 *[15]. Substitutions also varied widely between individual isolates. Similar results have been reported by Perea *et al*. who detected 13 *ERG11 *mutations in 20 *C. albicans *isolates with high level fluconazole resistance of which 11 were linked to resistance [5]. In contrast, just a single *ERG11 *mutation profile (comprising the same two mutations) was found in 14 of 15 fluconazole-resistant isolates in another study [17].

To our knowledge the G450V amino acid substitution has not been previously identified among isolates with reduced susceptibility to azoles. Most of the other substitutions described here have previously been seen in azole-resistant isolates [5,15,17,20] In particular, the substitutions G464S, G307S and G448E, known to confer azole resistance [5,12,15], were identified in three or more isolates. However, it is notable that the substitutions Y132H, S405F and R467K which appear to be prevalent in the United States and Europe were rare in Australian isolates [5,12,13,15]. Nineteen of the 20 amino acid substitutions, including G450V, present in the test isolates were clustered into the three "hot-spot" regions as described previously [19]. These hot spots include the residues 105–165 near the N-terminus of the protein, region 266–287 and region 405–488 located towards the C terminus of the protein. The exception was the G307S substitution (n = 3 isolates). However, in a computer-generated model of Erg11p, G307S is located close to the heme cofactor binding site. As such, substitutions at this residue might be expected to impact negatively on the binding of the azole [28].

In contrast to the fluconazole-resistant strains described above, 22% of fluconazole-susceptible isolates contained no *ERG11 *mutations and of those that did, substantially fewer (five compared with 20) amino acid substitutions were detected. Also of interest, all Erg11p amino acid substitutions from isolates with reduced azole susceptibility phenotypes were homozygous whereas with one exception (E266D), those in fluconazole-susceptible isolates were present as heterozygous substitutions. While these two observations support the general notion that *ERG11 *mutations are linked to azole resistance, the presence of *ERG11 *mutations in susceptible isolates is not readily explained. Development of "resistance" requires prolonged exposure to an azole [3,4]; however previous studies have not attempted to relate mutations in susceptible isolates to fluconazole exposure. Due to the retrospective nature of the present study we were unable to test this association.

The limitations of this study are recognised. Given the small numbers of isolates in our collection and that the presence of *ERG11 *mutations are not necessarily functionally related to resistance, we were unable to determine the clinical relevance of the *ERG11 *mutations identified. Since the substitutions E266D, D116E and V347I were present in both fluconazole-susceptible and, resistant isolates, it could be argued that they are unlikely to have contributed to reduced azole susceptibility [5,12,17,19]. On the other hand, with one exception, all identified mutations were heterozygous in fluconazole-susceptible isolates; the finding supports the contention that loss of heterozygosity in a diploid species such as *C. albicans *is a step in the development of the azole-resistant phenotype [3,20,29]. It is also possible that many *ERG11 *polymorphisms whilst not conferring resistance *per se*, may play a role in increasing the level of resistance [12,21].

Conversely, the absence of substitutions G307S, G448E, G464S, Y132H, S405F and R467K, in susceptible isolates strongly suggests they have contributed to the resistant phenotype. This hypothesis can be tested by site-directed mutagenesis and expression studies of specific *ERG11 *alleles in *Saccharomyces cerevisiae*. Using this approach, Sanglard and co-workers demonstrated that the substitutions G464S, Y132H, S405F and R467K were linked to azole resistance among their collection of isolates [12]; similar studies are warranted to determine if the new substitution G450V is associated with resistance. Testing matched, susceptible and resistant, isolates from the same patient for *ERG11 *mutations may also assist in determining if particular mutations impact on azole resistance; unfortunately, matched isolates were not available in the present study. In general, neither the type or number of mutations in isolates sequentially obtained from the same patient correlated with azole MICs (Table 2), emphasising the need to assess additional genes to understand the contribution of each to the resistance phenotype. As such, methods that detect polymorphisms are well-placed to screen large numbers of isolates from different sources for mutations and to guide functional testing of these isolates for resistance.

This study demonstrates a new application of a simple RCA-based technique for the rapid and accurate detection of SNPs in the *ERG11 *gene as potential markers of resistance and for the tracking of resistant strains. Other sequencing-independent methods include conventional real time PCR and/or other probe-based technologies eg. molecular beacons or TaqMan probes [30,31]. Results using conventional real time PCR are well-known to be highly-dependent on the physical characteristics of the platform. Molecular beacons and TaqMan probe methods are conveniently available in the form of commercial kits. Although able to detect SNPs with good sensitivity [30,31], strict attention to the *Tm *of the probes is required to ensure adequate specificity. The RCA-based method described here offers several advantages over other amplification techniques in that ligation of the probe ends by DNA ligase requires perfectly-matched target-probe complexes preventing nonspecific amplification generated by conventional PCR and resulting in very high specificity. It is also rapid (2 h compared to 1–2 days for DNA sequencing following DNA extraction). Whilst the set-up costs of the assay are relatively high, (AUD 300 per probe), a typical commercial batch of each probe provides sufficient material for up to 5000 assays. Running costs are estimated at no more than AUD 2 per assay compared to AUD 15 for DNA sequencing. The limitations of RCA in the primary identification of resistance are acknowledged (see above). However, the technique is well-suited as an epidemiological tool for high throughput screening for commonly-encountered *ERG11 *SNPs to assist in the detection of potentially-resistant strains and to track the movement of such strains. Further, its utility in detecting SNPs in other genes that have been linked to azole resistance in *C. albcians *such as those encoding for the transcriptional activator of *CDR1 *(*TAC1*) and the transcriptional activator Upc2 (*UPC2*) [32,33] warrant consideration.

## Conclusion

In conclusion, the sensitive and specific RCA-based assay proved to be a simple robust method for the rapid detection of *ERG11 *mutations and showed excellent concordance with DNA sequencing. It has good potential as a tool for tracking specific strains and identifying markers/co-markers of azole resistance. Broader implications include application of the method in the study of oher gene mutations linked to azole resistance in *C. albicans *and of azole resistance in other fungi such as *Aspergillus fumigatus *in which *ERG11 *mutations are a major mechanism of resistance [34,35].

## Methods

### *C. albicans *isolates

Eight fluconazole-resistant "reference" isolates with previously-described mutations in *ERG11 *(strains C438, C440, C470, C480, C507, C527, C577 and C594 provided by A. Chau, Schering-Plough Research Institute, Kenilworth, New Jersey; Table 1) [15] were used to validate the RCA assay. Two fluconazole-susceptible isolates (strains ATCC 10231 and ATCC 90028) were purchased from the American type culture collection (ATCC; Rockville, Md). Of 46 Australian clinical *C. albicans *isolates, 25 (obtained from 19 patients) were resistant, or had reduced susceptibility to fluconazole (five patients – patient 3, 6, 8, 12 and 16 had >1 isolate recovered on separate occasions) and 21 were fluconazole-susceptible (Table 2). These isolates were from the culture collection of the Clinical Mycology laboratory, Westmead Hospital, Sydney and the Mycology Unit, Women's and Children's Hospital, Adelaide. The experimental work was approved as part of a Centre of Clinical Research Excellence Grant awarded by the National Health and Medical Research Council of Australia (grant #264625) and approved by the Scientific Advisory Committee, Sydney West Area Health Service and the Research and Development Committee, Centre for Infectious Diseases and Microbiology Laboratory Services, Westmead Hospital. Thus, 33 isolates with reduced fluconazole susceptibility and 23 fluconazole-susceptible isolates were studied. Isolates were identified as *C. albicans *by standard phenotypic methods [36] and maintained on Sabouraud's dextrose agar at 4°C until required.

### Broth microdilution susceptibility testing

MICs of fluconazole and voriconazole (Pfizer Australia, Ryde, Australia) were determined for the ATCC and Australian study isolates (total n = 48) by broth microdilution using the Clinical and Laboratory Standards Institute (CLSI) M27-A2 protocol for susceptibility testing of yeasts [37]. *Candida parapsilosis *ATCC 22019 and *Candida krusei *ATCC 6528 were the quality control strains for each test run. The MIC endpoint was the lowest concentration of drug resulting in 50% growth inhibition compared with growth in the control (drug-free) well. Isolates were categorised as susceptible (MIC ≤ 8 μg/ml), susceptible dose-dependent (S-DD; MIC 16–32 μg/ml) or resistant (MIC ≥ 64 μg/ml) to fluconazole according to CLSI methodology [37]. Fluconazole and voriconazole MICs for the "reference isolates" have been reported [15] (Table 1).

### DNA extraction and PCR amplification of the ERG11 gene

DNA extraction was performed as described previously [38]. The near-full length *ERG11 *gene (1480 bp) was amplified with primers ERG11-S (5' aggggttccatttgtttaca 3') and ERG11-A (5' ccaaatgatttctgctggtt 3'; Beijing AUGCT Biotechnology Co. Ltd., Beijing, China) preparatory to hybridization with padlock probes and subsequent RCA (all isolates; see below) and for *ERG11 *sequence analysis (ATCC and Australian isolates).

Each PCR reaction contained: 1.5 μl (12–15 ng/μl) template DNA, 0.25 μl (50 pmol/μl) each of forward primer and reverse primer, 1.25 μl dNTPs (2.5 mM of each dNTP; [Roche Diagnostics, Mannheim, Germany]), 0.1 μl HotStar *Taq *polymerase (5 units/μl), 2.5 μl 10 × PCR buffer, (Qiagen, Doncaster, Victoria, Australia) and water to a total volume of 25 μl. Amplification was performed on a Mastercycler gradient thermocycler (Eppendorf AG, North Ryde, Australia). The thermal cycling conditions were 95°C for 15 min, followed by 35 cycles of 94°C for 45 s, 58°C for 45 s, and 72°C for 90 s, with a final extension step at 72°C for 10 min. PCR product was visualised under UV illumination to verify amplicon quantity prior to sequence analysis or RCA.

### ERG11 sequence analysis

PCR products were purified using the PCR Product Pre-sequencing Kit (USB Corporation, Cleveland, Ohio USA) and sequenced using ERG11-S and ERG11-A primers, and the BigDye Terminator (version 3.1) cycle sequencing kit in the ABI PRISM 3100 genetic analyser (Applied Biosystems, Foster City, CA). Sequences were entered into a BLASTn sequence analysis search and analyzed using editing and analyses programs in the BioManager (ANGIS) facility (accessed via. http://angis.org.au/).

### Primer and padlock probe design

The *ERG11 *sequence of the azole-susceptible strain *C. albicans *ATCC 28526 as published by Marichal *et al*. (GenBank database accession no. AF153844) was used for probe design. This sequence was chosen because *C. albicans *ATCC 28526 has been extensively characterised. A total of 24 padlock probes targeting 24 different *ERG11 *mutation sites were designed (Additional file 1). Ten of the 24 probes were designed to detect 10 mutations present in the "reference" isolates (Table 1); the remaining 14 targeted additional *ERG11 *mutations which have been identified in azole-resistant isolates [5,10,15,19].

The probes were 106–123 nucleotides (nt) in length, consisting of two adjacent target complementary sequences with a 48 nt linker region (Figure 1). To optimise binding to target DNA, probes were designed with a minimum of secondary structure and with a Tm of the 5'-end probe binding arm greater than the temperature used for probe ligation (62°C; see below). To increase the specificity, the 3'-end binding arm was designed to have a Tm (51–56°C) below the ligation temperature [25]. In particular, careful attention was paid to the linker region for each point mutation-specific probe to (i) minimise similarity to those mutations closely-located to the mutation of interest and (ii) to allow primer binding during RCA and amplification of the probe-specific signal. The 2 primers used for RCA – RCA primer 1 (5' ATGGGCACCGAAGAAGCA 3', Tm 55°C) and RCA primer 2 (5' CGCGCAGACACGATA 3', Tm 55°C) – were designed to specifically bind the linker region of the probes (Additional file 1)

### Purification of RCA template

Prior to ligation of the probe, *ERG11 *PCR products were purified to remove excess buffer, dNTP and primers: 25 μl of the PCR product was added to a well of a Millipore PCR purification plate (Pall Life Sciences, Ann Arbor, MI, USA) which was then placed on a vacuum manifold for 10–20 min to draw fluid and small particles through the membrane, leaving DNA on top of the membrane. A further 25 μl of dH_2_O was added to the well and the process repeated. The plate was removed from the vacuum, 20 μl of dH_2_O was added and the mixture incubated at 25°C for 2 min before transferring to a clean Eppendorf tube. Purified PCR products were stored at 4°C.

### Ligation of padlock probe and exonucleolysis

Purified amplified PCR product (10^11 ^copy numbers of DNA template [DNA calculator; http://www.uri.edu/research/gsc/resources/cndna.html]) was mixed with 2 U of *Pfu *DNA ligase (Stratagene, La Jolla, CA, USA) and 0.1 μM padlock probe as previously described [25] and subjected to multiple cycle ligation comprising one cycle of denaturation at 94°C for 5 min, followed by five cycles at 94°C for 30 s and 4 min of ligation at 62°C. Exonucleolysis was then performed to remove unligated probe and template PCR product; the purpose of the last step is to reduce subsequent ligation-independent amplification events during RCA. It was performed in 20-μl volumes by adding 10 U each of exonuclease I and exonuclease III (New England Biolabs, UK) to the ligation mixture and incubating at 37°C for 60 min followed by 95°C for 3 min.

### RCA

After exonucleolysis, RCA reactions was performed in 50 μl volumes containing 8 U of Bst DNA polymerase (New England Biolabs), 400 μM deoxynucleotide triphosphate mix, 10 pmol of each RCA primer, 5% of dimethyl sulfoxide (v/v) and 10 × SYBR Green I (Sigma-Aldrich, Castle Hill, Australia). Probe signals were amplified by incubation at 65°C for 30 min and the accumulation of dsDNA products were monitored using a Corbett RotorGeneTM 6000 real-time PCR machine (Corbett Research, Mortlake, Australia). Probe signals were also visualised on a 1.5% agarose gel to verify the specificity of probe-template binding.

### Nucleotide sequence accession numbers

The *ERG11 *sequences of the study isolates have been deposited in the GenBank database with the following accession numbers: FJ159508, FJ159444 to FJ159507 inclusive and FJ232378 to FJ232396 inclusive.

## Authors' contributions

SCAC, FK, TCS and HW designed the research. HW and BW carried out the molecular work and sequence alignment. MX participated in the sequence alignment. NP, FW and DE carried out the microbiological identification and susceptibility experiments. PM helped draft the manuscript and performed the susceptibility work on the "reference" isolates. HW, FK, TCS, FW and SCAC wrote the manuscript. All authors approved the final version of the manuscript.

## Supplementary Material

Additional file 1 **Padlock probes and primers used for RCA**. The data provide the names and sequences of the probes and primers used in the study for RCA. Click here for file

## References

[B1] Eggimann P Garbino J Pittet D Epidemiology of Candida species infections in critically ill non-immunosuppressed patients Lancet Infect Dis 2003 3 685 702 10.1016/S1473-3099(03)00801-6 14592598

[B2] Odds FC Webster CE Mayuranathan P Simmons PD Candida concentrations in the vagina and their association with signs and symptoms of vaginal candidosis J Med Vet Mycol 1988 26 277 283 10.1080/02681218880000391 3236147

[B3] White TC Marr KA Bowden RA Clinical, cellular, and molecular factors that contribute to antifungal drug resistance Clin Microbiol Rev 1998 11 382 402 9564569 10.1128/cmr.11.2.382 PMC106838

[B4] Morschhauser J The genetic basis of fluconazole resistance development in Candida albicans Biochim Biophys Acta 2002 1587 240 248 12084466 10.1016/s0925-4439(02)00087-x

[B5] Perea S Lopez-Ribot JL Kirkpatrick WR McAtee RK Santillan RA Martinez M Calabrese D Sanglard D Patterson TF Prevalence of molecular mechanisms of resistance to azole antifungal agents in Candida albicans strains displaying high-level fluconazole resistance isolated from human immunodeficiency virus-infected patients Antimicrob Agents Chemother 2001 45 2676 2684 10.1128/AAC.45.10.2676-2684.2001 11557454 PMC90716

[B6] Rex JH Rinaldi MG Pfaller MA Resistance of Candida species to fluconazole Antimicrob Agents Chemother 1995 39 1 8 7695288 10.1128/aac.39.1.1 PMC162475

[B7] Lopez-Ribot JL McAtee RK Lee LN Kirkpatrick WR White TC Sanglard D Patterson TF Distinct patterns of gene expression associated with development of fluconazole resistance in serial Candida albicans isolates from human immunodeficiency virus-infected patients with oropharyngeal candidiasis Antimicrob Agents Chemother 1998 42 2932 2937 9797228 10.1128/aac.42.11.2932 PMC105968

[B8] Kelly SL Arnoldi A Kelly DE Molecular genetic analysis of azole antifungal mode of action Biochem Soc Trans 1993 21 1034 1038 8131893 10.1042/bst0211034

[B9] Franz R Kelly SL Lamb DC Kelly DE Ruhnke M Morschhauser J Multiple molecular mechanisms contribute to a stepwise development of fluconazole resistance in clinical Candida albicans strains Antimicrob Agents Chemother 1998 42 3065 3072 9835492 10.1128/aac.42.12.3065 PMC106000

[B10] Sanglard D Odds FC Resistance of Candida species to antifungal agents: molecular mechanisms and clinical consequences Lancet Infect Dis 2002 2 73 85 10.1016/S1473-3099(02)00181-0 11901654

[B11] White TC Increased mRNA levels of ERG16, CDR, and MDR1 correlate with increases in azole resistance in Candida albicans isolates from a patient infected with human immunodeficiency virus Antimicrob Agents Chemother 1997 41 1482 1487 9210670 10.1128/aac.41.7.1482 PMC163944

[B12] Sanglard D Ischer F Koymans L Bille J Amino acid substitutions in the cytochrome P-450 lanosterol 14alpha-demethylase (CYP51A1) from azole-resistant Candida albicans clinical isolates contribute to resistance to azole antifungal agents Antimicrob Agents Chemother 1998 42 241 253 9527767 10.1128/aac.42.2.241 PMC105395

[B13] White TC The presence of an R467K amino acid substitution and loss of allelic variation correlate with an azole-resistant lanosterol 14alpha demethylase in Candida albicans Antimicrob Agents Chemother 1997 41 1488 1494 9210671 10.1128/aac.41.7.1488 PMC163945

[B14] Favre B Didmon M Ryder NS Multiple amino acid substitutions in lanosterol 14alpha-demethylase contribute to azole resistance in Candida albicans Microbiology 1999 145 2715 2725 10537193 10.1099/00221287-145-10-2715

[B15] Chau AS Mendrick CA Sabatelli FJ Loebenberg D McNicholas PM Application of real-time quantitative PCR to molecular analysis of Candida albicans strains exhibiting reduced susceptibility to azoles Antimicrob Agents Chemother 2004 48 2124 2131 10.1128/AAC.48.6.2124-2131.2004 15155210 PMC415610

[B16] White TC Holleman S Dy F Mirels LF Stevens DA Resistance mechanisms in clinical isolates of Candida albicans Antimicrob Agents Chemother 2002 46 1704 1713 10.1128/AAC.46.6.1704-1713.2002 12019079 PMC127245

[B17] Xu Y Chen L Li C Susceptibility of clinical isolates of Candida species to fluconazole and detection of Candida albicans ERG11 mutations J Antimicrob Chemother 2008 61 798 804 10.1093/jac/dkn015 18218640

[B18] Lamb DC Kelly DE Schunck WH Shyadehi AZ Akhtar M Lowe DJ Baldwin BC Kelly SL The mutation T315A in Candida albicans sterol 14alpha-demethylase causes reduced enzyme activity and fluconazole resistance through reduced affinity J Biol Chem 1997 272 5682 5688 10.1074/jbc.272.15.9986 9038178

[B19] Marichal P Koymans L Willemsens S Bellens D Verhasselt P Luyten W Borgers M Ramaekers FC Odds FC Bossche HV Contribution of mutations in the cytochrome P450 14alpha-demethylase (Erg11p, Cyp51p) to azole resistance in Candida albicans Microbiology 1999 145 2701 2713 10537192 10.1099/00221287-145-10-2701

[B20] Lee MK Williams LE Warnock DW Arthington-Skaggs BA Drug resistance genes and trailing growth in Candida albicans isolates J Antimicrob Chemother 2004 53 217 224 10.1093/jac/dkh040 14688046

[B21] Akins RA An update on antifungal targets and mechanisms of resistance in Candida albicans Med Mycol 2005 43 285 318 10.1080/13693780500138971 16110776

[B22] Nilsson M Lock and roll: single-molecule genotyping in situ using padlock probes and rolling-circle amplification Histochem Cell Biol 2006 126 159 164 10.1007/s00418-006-0213-2 16807721

[B23] Nilsson M Dahl F Larsson C Gullberg M Stenberg J Analyzing genes using closing and replicating circles Trends Biotechnol 2006 24 83 88 10.1016/j.tibtech.2005.12.005 16378651

[B24] Wang B Potter SJ Lin Y Cunningham AL Dwyer DE Su Y Ma X Hou Y Saksena NK Rapid and sensitive detection of severe acute respiratory syndrome coronavirus by rolling circle amplification J Clin Microbiol 2005 43 2339 2344 10.1128/JCM.43.5.2339-2344.2005 15872263 PMC1153787

[B25] Kong F Tong Z Chen X Sorrell T Wang B Wu Q Ellis D Chen S Rapid identification and differentiation of Trichophyton species, based on sequence polymorphisms of the ribosomal internal transcribed spacer regions, by rolling-circle amplification J Clin Microbiol 2008 46 1192 1199 10.1128/JCM.02235-07 18234865 PMC2292936

[B26] Zhou X Kong F Sorrell TC Wang H Duan Y Chen SC Practical method for detection and identification of Candida, Aspergillus, and Scedosporium spp. by use of rolling-circle amplification J Clin Microbiol 2008 46 2423 2427 10.1128/JCM.00420-08 18495860 PMC2446935

[B27] Reference method for broth dilution antifungal susceptibility testing of yeasts. Approved standard NCCLS document M27-A3 2008 3 National Committee for Clinical Laboratory Standards: Wayne, PA

[B28] Xiao L Madison V Chau AS Loebenberg D Palermo RE McNicholas PM Three-dimensional models of wild-type and mutated forms of cytochrome P450 14alpha-sterol demethylases from Aspergillus fumigatus and Candida albicans provide insights into posaconazole binding Antimicrob Agents Chemother 2004 48 568 574 10.1128/AAC.48.2.568-574.2004 14742211 PMC321559

[B29] Asai K Tsuchimori N Okonogi K Perfect JR Gotoh O Yoshida Y Formation of azole-resistant Candida albicans by mutation of sterol 14-demethylase P450 Antimicrob Agents Chemother 1999 43 1163 1169 10223930 10.1128/aac.43.5.1163 PMC89127

[B30] Yesilkaya H Meacci F Niemann S Hillemann D Rusch-Gerdes S Barer MR Andrew PW Oggioni MR Evaluation of molecular-Beacon, TaqMan, and fluorescence resonance energy transfer probes for detection of antibiotic resistance-conferring single nucleotide polymorphisms in mixed Mycobacterium tuberculosis DNA extracts J Clin Microbiol 2006 44 3826 3829 10.1128/JCM.00225-06 17021120 PMC1594805

[B31] Gibson NJ The use of real-time PCR methods in DNA sequence variation analysis Clin Chim Acta; Int J Clin Chem 2006 363 32 47 10.1016/j.cccn.2005.06.022 16182268

[B32] Coste A Turner V Ischer F Morschhauser J Forche A Selmecki A Berman J Bille J Sanglard D A mutation in Tac1p, a transcription factor regulating CDR1 and CDR2, is coupled with loss of heterozygosity at chromosome 5 to mediate antifungal resistance in Candida albicans Genetics 2006 172 2139 2156 10.1534/genetics.105.054767 16452151 PMC1456413

[B33] MacPherson S Akache B Weber S De Deken X Raymond M Turcotte B Candida albicans zinc cluster protein Upc2p confers resistance to antifungal drugs and is an activator of ergosterol biosynthetic genes Antimicrob Agents Chemother 2005 49 1745 1752 10.1128/AAC.49.5.1745-1752.2005 15855491 PMC1087678

[B34] Mellado E Garcia-Effron G Alcazar-Fuoli L Melchers WJ Verweij PE Cuenca-Estrella M Rodriguez-Tudela JL A new Aspergillus fumigatus resistance mechanism conferring in vitro cross-resistance to azole antifungals involves a combination of cyp51A alterations Antimicrob Agents Chemother 2007 51 1897 1904 10.1128/AAC.01092-06 17371828 PMC1891382

[B35] Garcia-Effron G Dilger A Alcazar-Fuoli L Park S Mellado E Perlin DS Rapid detection of triazole antifungal resistance in Aspergillus fumigatus J Clin Microbiol 2008 46 1200 1206 10.1128/JCM.02330-07 18234874 PMC2292958

[B36] Warren N Hazen K Murray RPBE, Pfaller MA, Tenover FC, Yolken RH Candida, Cryptococcus, and other yeasts of medical importance Manual of Clinical Microbiology 1999 Washington, D.C.: ASM Press 1184 1199

[B37] Reference method for broth dilution antifungal susceptibility testing of yeasts. Approved standard NCCLS document M27-A3 2002 3 National Committee for Clinical Laboratory Standards: Wayne, PA

[B38] Playford EG Kong F Sun Y Wang H Halliday C Sorrell TC Simultaneous detection and identification of Candida, Aspergillus, and Cryptococcus species by reverse line blot hybridization J Clin Microbiol 2006 44 876 880 10.1128/JCM.44.3.876-880.2006 16517870 PMC1393088

