# Supplementary material for: Rapid detection of ERG11 gene mutations in clinical Candida albicans isolates with reduced susceptibility to fluconazole by rolling circle amplification and DNA sequencing
Source: BMC Microbiol. 2009 Aug 14;9:167. doi: 10.1186/1471-2180-9-167 (PMC2782262; doi:10.1186/1471-2180-9-167)
Supplement: Additional file 1 — Padlock probes and primers used for RCA. The data provide the names and sequences of the probes and primers used in the study for RCA. [file 1471-2180-9-167-S1.doc]

**ADDITIONAL FILES**

**Additional File 1: ­Padlock probes and primers used for RCA**

| **Probe or primer** | Target *ERG11* mutation | **GenBank accession no.** | **Sequence and locations of the two binding arms on comparison with relevant reference GenBank sequences (5’-3’) or sequence of primer^a^** |
| --- | --- | --- | --- |
| ***Probes targerting mutations in “reference isolates”*** | | | |
| Ca-A61V | C182T | AF153844 | | | **^a^**p-181cagaaccaaaccaaggaatccaa159 | | --- | | | --- | --- |   gatca^b^*TGCTTCTTCGGTGCCCAT*aatgccacgttaacagtcag*CGCGCAGACACGATA*gtcta  200tgttgaccatatgaagcta182 |
| Ca-G307S | G919A | AF153844 | **^a^**p-918cataagaataccaattaaaagattagcaatttcttg883  gatca*TGCTTCTTCGGTGCCCAT*gactctcgctcgacacagtag*CGCGCAGACACGATA*gtcta  937cagaagtatgttgaccact919 |
| Ca-G450E | G1349A | AF153844 | **^a^**p-1348caaacccataatcaacttcatcagaag1322 |
|  |  |  | gatca*TGCTTCTTCGGTGCCCAT*tgaccgtgctatgaatgcat*CGCGCAGACACGATA*gtcta |
|  |  |  | 1366cccctttagaaactttct1349 |
| Ca-G464S | G1390A | AF153844 | **^a^**p-1389aaatggtaaataaggtgaagaaacccc1363 |
|  |  |  | gatca*TGCTTCTTCGGTGCCCAT*gctaacctggtaccgtcatt*CGCGCAGACACGATA*gtcta |
|  |  |  | 1409catctatgtctaccaccact1390 |
| Ca-H283R | A848G | AF153844 | **^a^**p-847GaatCAATAAGGAATCAATTAAATCACGA819 |
|  |  |  | gatca*TGCTTCTTCGGTGCCCAT*gactctcgctcgacacagtag*CGCGCAGACACGATA*gtcta |
|  |  |  | 868caCCATCTTTATAAGTTGAAC848 |
| Ca-K128T | A383C | AF153844 | **^a^**p-382taccgaaaactggagtagttaaatgtttataagc349 |
|  |  |  | gatca*TGCTTCTTCGGTGCCCAT*gcttagcttggcatgtcact*CGCGCAGACACGATA*gtcta |
|  |  |  | 401caatcataaataacccctg383 |
| Ca-R467I | G1400T | AF153844 | **^a^**p-1339taccaccaccaaatggtaaataaggt1374  gatca*TGCTTCTTCGGTGCCCat*cctactagttgcacgctgttc*CGCGCAGACACGATA*gtcta  1418tccccaatacatctatgta1400 |
| Ca-S405F | C1214T | AF153844 | **^a^**p-1213aaactaaaacgtaatgaccttttggaaca1185 |
|  |  |  | gatca*TGCTTCTTCGGTGCCCAT*tcgtggctagtcgaatcttag*CGCGCAGACACGATA*gtcta |
|  |  |  | 1230atgagcataacctggaa1214 |
| Ca-Y132H | T394C | AF153844 | **^a^**p-393aataacccctttaccgaaaactgg370 |
|  |  |  | gatca*TGCTTCTTCGGTGCCCAT*tacgaggtgcggatagctac*CGCGCAGACACGATA*gtcta  412tagaatttggacaatcatg394 |
| Ca-Y257H | T769C | AF153844 | **^a^**p-768agtagcagagattttcttttgagcagc742 |
|  |  |  | gatca*TGCTTCTTCGGTGCCCAT*cctagatcagacgttcctgtc*CGCGCAGACACGATA*gtcta  790TCAGTTTAATTTCTTTCATATG769 |
| ***Probes targeting other ERG11 mutations*** | | | |
| Ca-D116E | T348A | AF153844 | **^a^**p-347TCTTCAGCAGAAACATCAGATAATTTAGC319  gatca*TGCTTCTTCGGTGCCCAT*aacgactccaggttagcctag*CGCGCAGACACGATA*gtcta  370GAGTAGTTAAATGTTTATAAGCT348 |
| Ca-D278E | T834A | AF153844 | **^a^**p-833TCAATTAAATCACGATTTGGATCAATATC805  gatca*TGCTTCTTCGGTGCCCAT*cctactagttgcacgctgttc*CGCGCAGACACGATA*gtcta  852TGAATGAATCAATAAGGAT834 |
| Ca-E266D | A798C | AF153844 | **^a^**p-797tctcttctcagtttaatttctttcatataagtagca762  gatca*TGCTTCTTCGGTGCCCAT*gactctcgctcgacacagtag*CGCGCAGACACGATA*gtcta  814gatcaatatcaccacgg798 |
| Ca-F105L | T313C | AF153844 | **^a^**p-312AACAAATTCATGACCTTTTGGACC289  gatca*TGCTTCTTCGGTGCCCAT*gactctcgctcgacacagtag*CGCGCAGACACGATA*gtcta  333ATCAGATAATTTAGCATTGAG313 |
| Ca-F145L | T433C | AF153844 | **^a^**p-432TTTTTTTTGTTCCATTAATCTAGAATTTGG403  gatca*TGCTTCTTCGGTGCCCAT*gcttagcttggcatgtcact*CGCGCAGACACGATA*gtcta  444AAAGCAAATTTAGCAAG433 |
| Ca-F449S | T1346C | AF153844 | **^a^**p-1345ACCCATAATCAACTTCATCAGAAGAGTT1318  gatca*TGCTTCTTCGGTGCCCAT*gactctcgctcgacacagtag*CGCGCAGACACGATA*gtcta  1363CTTTAGAAACTTTCCCAG1346 |
| Ca-G448E | G1343A | AF153844 | **^a^**p-1342cataatcaacttcatcagaagagttaaatgaaa1310  gatca*TGCTTCTTCGGTGCCCAT*gctaacctggtaccgtcatt*CGCGCAGACACGATA*gtcta  1360TAGAAACTTTCCCAAACT1343 |
| Ca-G448V | G1343T | AF153844 | **^a^**p-1342cataatcaacttcatcagaagagttaaatgaaa1310  gatca*TGCTTCTTCGGTGCCCAT*gactctcgctcgacacagtag*CGCGCAGACACGATA*gtcta  1360TAGAAACTTTCCCAAACA1343 |
| Ca-G465S | G1393A | AF153844 | **^a^**p-1392ACCAAATGGTAAATAAGGTGAAGAAACC1365  gatca*TGCTTCTTCGGTGCCCAT*aatgccacgttaacagtcag*CGCGCAGACACGATA*gtcta  1414CAATACATCTATGTCTACCACT1393 |
| Ca-K143R | A428G | AF153844 | **^a^**p-427tttgttccattaatctagaatttggacaat398  gatca*TGCTTCTTCGGTGCCCAT*gactctcgctcgacacagtag*CGCGCAGACACGATA*gtcta  446gcaaatttagcaaattttc428 |
| Ca-K287R | A860G | AF153844 | **^a^**p-859tataagttgaatgaatcaataaggaatcaattaaat824  gatca*TGCTTCTTCGGTGCCCAT*gactctcgctcgacacagtag*CGCGCAGACACGATA*gtcta  877TCATTTTCACACCATCTC860 |
| Ca-R467K | G1400A | AF153844 | **^a^**p-1399taccaccaccaaatggtaaataaggt1374  gatca*TGCTTCTTCGGTGCCCAT*tgaccgtgctatgaatgcat*CGCGCAGACACGATA*gtcta  1417ccccaatacatctatgtT1400 |
| Ca-V437I | G1309A | AF153844 | **^a^**p-1308agaattggctttagcagcagca1287  gatca*TGCTTCTTCGGTGCCCAT*gactctcgctcgacacagtag*CGCGCAGACACGATA*gtcta  1329atcagaagagttaaatgaaat1309 |
| Ca-V488I | G1462A | AF153844 | **^a^**p-1461aaaagtagttaaaatggttcctaattgaacataag1427  gatca*TGCTTCTTCGGTGCCCAT*gactctcgctcgacacagtag*CGCGCAGACACGATA*gtcta  1486caatagtccatcttaaattataaat1462 |
| ***RCA primer*** | | | |
| RCA Primer 1^b^ |  |  | ATGGGCACCGAAGAAGCA^b^ |
| RCA Primer 2^c^ |  |  | CGCGCAGACACGATA^c^ |

a Refers to the 5’-end of probe: “p-“ indicates 5’ phosphorylation. The sequences of the 5’ and 3’ binding arms of the probes (derived from reference GenBank sequence) are underlined; these are joined by the backbone of the probe including the non-specific linker region where the sequence is shown in lower case.

^b^ Binding site of RCA primer 1 to the padlock probe, generating a long single-stranded DNA. The sequence of RCA primer 1 is the complement of the segments, in reverse, represented by the underlined italicized nucleotides.

^c^ Binding site of RCA primer 2. This primer binds to nascent single-stranded DNAs as their binding sites become available. Its sequence is the same as that of the segments shown in italics.
